# Effects of Single-Dose Prucalopride on Intestinal Hypomotility in Horses: Preliminary Observations

**DOI:** 10.1038/srep41526

**Published:** 2017-01-27

**Authors:** Fulvio Laus, Margherita Fratini, Emanuele Paggi, Vanessa Faillace, Andrea Spaterna, Beniamino Tesei, Katia Fettucciari, Gabrio Bassotti

**Affiliations:** 1 School of Bioscences and Veterinary Medicine, University of Camerino, Via Circonvallazione 63/95, 62024 Matelica, Italy; 2 Department of Experimental Medicine, Perugia University School of Medicine, Piazzale Lucio Severi, 1, 06159 San Sisto (Perugia), Italy; 3 Department of Medicine, Perugia University School of Medicine, Piazzale Lucio Severi, 1, 06159 San Sisto (Perugia), Italy

## Abstract

Abnormalities of gastrointestinal motility are often a challenge in horses; however, the use of prokinetic drugs in such conditions must be firmly established yet. For this reason we carried out a preliminary study on the effects of prucalopride on intestinal motor activity of horses with gut hypomotility. The effect of prucalopride per os by oral dose syringe (2 mg/100 kg body weight) was assessed by abdominal ultrasound (evaluating duodenal, cecal, and colonic motor activity) in six horses with gut hypomotility. After administration of prucalopride, a significant increase of contractile activity was found in the duodenum at 30 minutes (p = 0.0005), 60 minutes (p = 0.01) and 90 minutes (p = 0.01), whereas in the cecum and in the left colon the increase was only present at 60 minutes (p = 0.03, and p = 0.02, respectively). No changes from baseline heart and respiratory rate or behavior side effects were observed after administration of the drug and throughout the observation period. Prucalopride may be a useful adjunct to the therapeutic armamentary for treating hypomotile upper gut conditions of horses. Dosing information is however needed to establish its actual clinical efficacy and its proper effects on the large bowel in these animals.

Gastro-intestinal motility abnormalities in horses can be due to several conditions including equine grass sickness, gastroduodenal ulceration, intraluminal obstruction or impaction, excessive wall distention, strangulating obstruction, peritonitis, duodenitis, proximal jejunitis, colitis, and postoperative ileus (POI)^1^. The latter is responsible for up to 86% equine deaths following abdominal surgery^2^.

Although several prokinetic drugs can be used in horses with gastrointestinal hypomotility, the use of these drugs is not well established yet^3^; bethanecol and neostigmine are cholinomimetic drugs that can be administered to horses, but are gravated by cholinergic side effects^1^, metoclopramide acts as a 5-hydroxytryptamine 4-receptor (5HT-4) agonist, 5HT-3 receptor antagonist, dopamine 1 (DA_1_) and 2 (DA_2_) receptors antagonist^1^, and domperidone as a competitive antagonist at peripheral DA_2_ receptors, even though his efficacy in horses needs to be further investigated^4^. Other drugs with proven prokinetic effect in horses include erythromycin, naloxone and, in horses affected by POI, lidocaine administered as a starting dose of 1.3 mg/kg followed by 0.05 mg/kg/min for 24 hours^1^. Cisapride is a second-generation benzamide that acts as a 5HT-4 agonist and 5HT-3 receptor antagonist; it can be used in managing persistent large colon impaction in horses, equine grass sickness, and as a preventative for POI^1^. However, due to serious cardiac side effects in humans this drug has been withdrawn from the market^5^ and it is presently difficult or impossible to find on the market, even for veterinary purposes.

Prucalopride is a dihydro-benzofurancarboxamide derivative approved in 2009 by the European Medicines Agency for the treatment of constipation in women^6^. Due to its selective high-affinity agonist effect for the serotonin receptor 5-HT4, prucalopride stimulates gut motility and secretion via 5-HT4 receptor activation through enhanced release of acetylcholine^7^,^8^. Of interest, oral administration of the drug causes a rapid absorption from the gastrointestinal tract, with very high bioavailability (>93%) that is not affected by food intake^6^,^9^. Although presently available for treatment of constipation, the available literature suggests that the enterokinetic properties of prucalopride may not be limited to the colon, but also involve the esophagus, the stomach, and the small bowel^10^.

Unfortunately, data on the effects of prucalopride as enterokinetic in horses are limited to one *in vitro* study, showing that this drug failed to elicit an increase of submaximal electrical field stimulation-induced contractions in smooth muscle strips sampled from horses^11^. On the other hand, the presence of 5-HT4 receptors in the equine gut and the effectiveness of other selective 5-HT4 agonists has been repeteadly demonstrated^12^,^13^,^14^. To the best of our knowledge, no report on prucalopride administration in horses is available in literature, and in the present paper, the prokinetic effect of was tested *in vivo* for the first time in horses with intestinal hypomotility.

## Methods

Six female standardbred horses (age 11 to 19, mean 15.0 ± 3.2 (SD) years, body weight from 383 to 512 Kg (mean 458.2 ± 53.8 Kg)) presenting clinical manifestation of gastrointestinal diseases (inappetence, poor body condition, weight loss, mild colic) were referred to the Veterinary Teaching Hospital, School of Biosciences and Veterinary Medicine, University of Camerino.

All horses were affected by primary equine squamous gastric disease (ESGD) graded by gastroscopy as grade III or IV, according to the criteria proposed by Sykes and colleagues^15^. Hypomotility was assessed by auscultation and ultrasonography (see below) performed daily and did not improve after administration of metoclopramide (0.1 mg/kg subcutaneously every 8 hours for 48 hours). It was therefore decided to use prucalopride.

**Figure 1:**
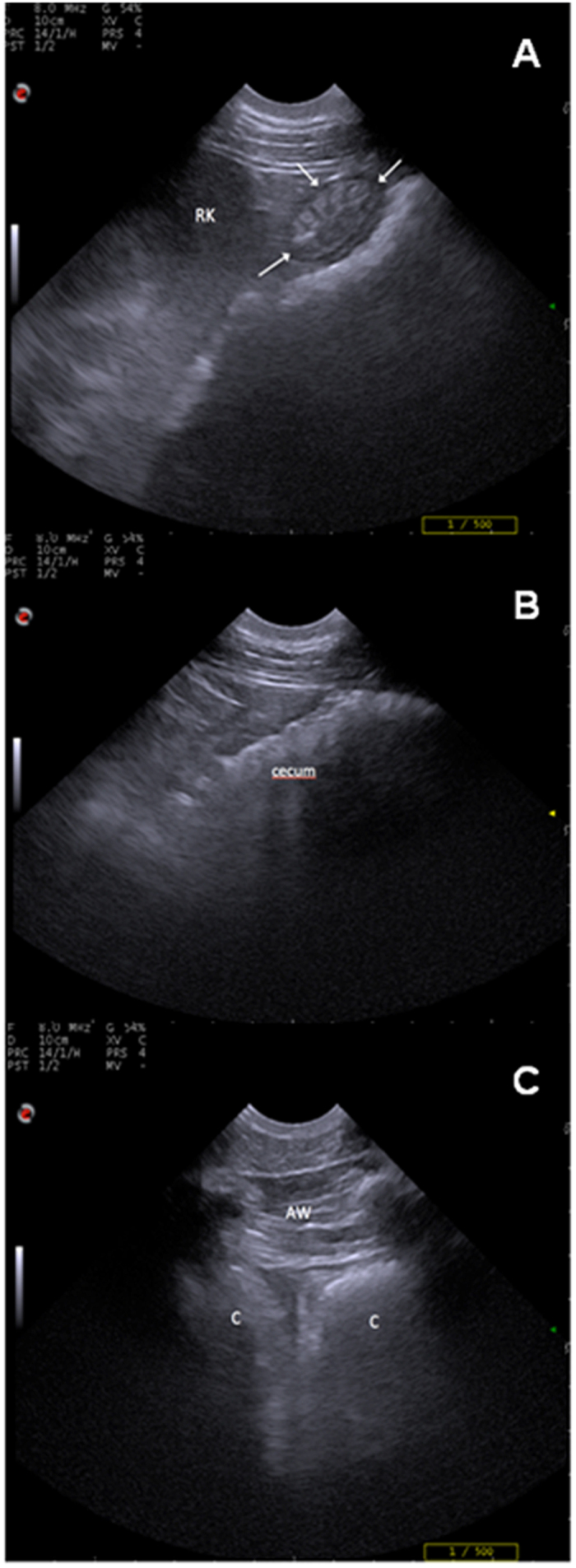
(**A**) ultrasonographic visualization of the descending duodenum (arrows). RK: right kidney. (**B**) ultrasonographic visualization of cecum. (**C**) ultrasonographic visualization of left ascending colon (**C**) with typical sacculation. AW: abdominal wall.

Motility of the descending duodenum, cecum, and left ascending colon (Fig. 1A–C) was assessed by ultrasonography, as previously described^16^,^17^. The three areas were clipped and cleaned with alcohol to obtain images as good as possible. Ultrasonography was performed with a curvilinear transducer at a frequency of 5 MHz (MyLabOne, Esaote, Italy). Briefly, the descending duodenum was imaged on the right side, from the 8^th^ or 18^rd^ intercostal space, along the line joining the olecranon and the tuber coxae, medially to the right lobe of the liver. The cecum was imaged in the dorsal part of the right paralumbar region. The left colon is normally viewed on all left wall but an acoustic window on the paralumbar region at level of the stifle was used.

Monitoring was started in the early afternoon, after the meal; a single dose of prucalopride (2 mg/100 kg body weight by oral dose syringe) was administered 40 minutes after the end of the meal and peristalsis was monitored before administration (basal) and at 5, 15, 30, 60, 90, 120, 180 and 240 minutes after administration of the drug. Water was available *ad libitum*. The drug dose was adapted from human studies, with this dose being a good compromise between effective pharmacologic action and paucity of side effects^6^.

The number of circular contraction in a cross sectional visualization was counted to assess duodenal motility. Deviation of cecal wall from the transducer was used to assess cecal contractions. For the left ascending colon changes in sacculation were considered, according to previously reported criteria^17^. For each anatomical location, intestinal contractions were counted during a 3-minute period.

Concerning clinical monitoring, behavioral patterns, heart and respiratory rates were assessed for every time-point used for ultrasonography (basal to 240 minutes).

All procedures were carried out in accordance with relevant guidelines and regulations and the experimental protocol was approved by the Institutional Animal Care and Use Committee of Camerino University (Protocol number 2/2016). All the horses’ owners gave written informed consent before enrollment.

Data analysis was carried out by means of the computer software program WINPEPI (PEPI-for-Windows)^18^. Mean and standard deviations were calculated for each measurement of contraction pre- and post- administration of prucalopride. Differences in intestinal motility (number of contractions) among the time-points was evaluated by the ANOVA for repeated measures with Bonferroni’s correction, and the Student’s t-test with multiple comparisons with the basal value after assessment of normal distribution by means of the Shapiro-Wilk test. Values of p < 0.05 were chosen for rejection of the null hypothesis.

## Results

**Figure 2:**
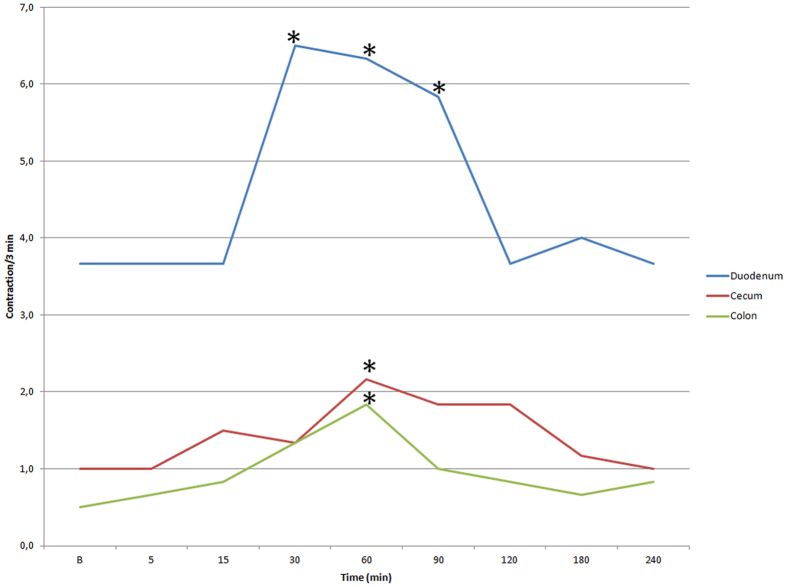
Ultrasonographic contractions recorded in the duodenum, cecum and colon in horses before (B = baseline) and after administration of 2 mg/100 Kg prucalopride. *statistically significant compared to B.

The overall results (expressed as mean ± SD) of contraction measurement in the three intestinal tract examined are reported in Table 1 and in Fig. 2.

Analysis of variance was statistically significant in the duodenum (F = 101.12, p < 0.0001) and in the cecum (F = 21.97, p = 0.002), but not in the colon (F = 1.77, p = 0.21), and the single comparisons with the basal values revealed significant differences at 30 minutes, (p = 0.0005), 60 minutes (p = 0.01) and 90 minutes (p = 0.01) for the descending duodenum, and only at 60 minutes for the cecum and the left colon (p = 0.03, and p = 0.02, respectively).

No signs of behavioral abnormality were registered during the study and no changes from baseline heart or respiratory rates were observed (Table 2).

## Discussion

Due to possible species-related different intestinal sensitivity to 5-HT agonists, data on characterization and activity of these receptors in horses are to date conflicting.

For instance, stimulatory effects of 5-HT that were reduced by 5-HT3 and 5-HT4 receptor antagonists have been reported on circular and longitudinal muscle of equine ileum and pelvic flexure^12^, and a widespread 5-HT-4 receptor immunoreactivity has been observed in all intestinal smooth muscle layers of duodenum, ileum and pelvic flexure sampled from healthy horses^14^. Moreover tegaserod, a 5-HT4 receptor agonist, stimulated large intestinal motility of horses in both *in vivo*^13^ and *in vitro*^12^,^19^, and increased the amplitude of smooth muscle contractions in a concentration-dependent manner on tissue preparations from duodenum and pelvic flexure sampled from healthy horses^14^. Another specific 5-HT4 agonist (mosapride) also increased myoelectric activity of equine jejunum *in vivo*^20^.

On the other hand, Delesalle and colleagues suggested that 5-HT receptors are not involved in contractile effect of serotonine in jejunum^21^, and in a recent preliminary report the same authors failed to find evidence for the presence of 5-HT4 receptors on the cholinergic neurons of the equine small intestine^11^. Other authors showed that the *in vitro* effects of 5-hydroxytryptamine (5-HT) and cisapride on the smooth muscle of horse are not altered by 5-HT4 antagonists, suggesting a primary non-cholinergic effect^22^, and that cisapride may modulate intestinal motility through a stimulation of nonserotonergic receptors^23^.

However, regardless of the mechanisms modulating intestinal contractions by means of 5-HT agonists, this class of drugs has proven to be active on intestinal motility of several species, including horses^24^,^25^,^26^.

Since prokinetic drugs are commonly used to treat intestinal dysmotilities in horses^1^,^3^,^27^, and the available data on current therapeutic approaches are (at least for some pathological conditions) still inconclusive^28^,^29^, there is the need for more targeted therapeutic approaches. Thus, in this preliminary study we evaluated the effects of prucalopride, a drug with proven intestinal prokinetic effects in humans and other animal species^6^,^7^, on intestinal motility of horses with gut hypomotility.

Our results showed that, 30 to 90 minutes after oral administration of prucalopride 2 mg/100 kg body weight, horses with postprandial intestinal hypomotility displayed an increased gut motor activity, maximal in the duodenum, with only a minimal increase in the cecum and the left colon. Although we cannot explain these differences in the various tracts of the gastrointestinal system, it is possible that different receptor expression or different mechanism between intestinal segments may play a role. Of interest, although it is unknown whether enterocytes or other cells in the equine duodenum are equipped with 5-HT4 receptors, luminal application of prucalopride may have motor effects due to 5-HT4 receptors on epithelial cells (possibly enteroendocrine cells but also other epithelial cells)^8^. Alternatively, it is possible that the dose of the drug was too small to exert more important stimulatory effects on the large bowel. Thus, even though this was a pilot trial conducted on a limited sample size, we feel these effects might be of clinical interest, and suggest that prucalopride could be tested in animals suffering from other forms of impaired motility such as intestinal impaction or postoperative ileus. The horses included in this study were in fact suffering from a chronic disease, for which it was not possible to have an immediate feedback on the improvement of clinical symptoms. However, prokinetics may be indicated to prevent colics that can be caused by hypomotility in horses affected by gastric ulceration^15^. Our results indicate that prucalopride could be used as supplement of specific therapy in horses affected by severe gastroduodenal ulceration causing intestinal hypomotility.

However, it must be taken into account the limit represented by the oral formulation of the drug, that cannot be always used, especially in horses with gastric reflux and in case of postoperative ileus. Moreover, the effects of repeated administrations of the drug are not known, and could provide insightful information on the actual propulsive effects of prucalopride-induced contractions in horses.

In this study we used ultrasonography to evaluate intestinal motor activity. Equine gastrointestinal motility is still poorly known, and it has been investigated using several techniques, including tissue culture, immunohistochemistry, molecular biology, electrophysiology and whole animal studies^30^. The use of ultrasonography in this setting represents an important investigative tool, since it is non-invasive and it allows repeated measurements in the time course with objective measurements of intestinal contractions^30^.

In conclusion, in this preliminary study we have shown that a single-dose oral administration of prucalopride is able to improve upper postprandial intestinal motility in horses with gut hypomotility. Further studies, possibly also with dose-finding trials and randomized controlled conditions, are needed to establish whether prucalopride has prokinetic effects also on the equine large bowel, and whether this drug could be added to the therapeutic armamentarium of treatments for intestinal hypomotility in horses.

## Additional Information

**How to cite this article**: Laus, F. *et al*. Effects of Single-Dose Prucalopride on Intestinal Hypomotility in Horses: Preliminary Observations. *Sci. Rep.* **7**, 41526; doi: 10.1038/srep41526 (2017).

**Publisher's note:** Springer Nature remains neutral with regard to jurisdictional claims in published maps and institutional affiliations.

## Figures and Tables

**Table 1:** Mean and standard deviation of number of contraction/3 min of the examined tracts at different time-points; SD = standard deviation; *significant differences with basal value (B).

Horse	duodenum									cecum									colon								
Time	B	5	15	30	60	90	120	180	240	B	5	15	30	60	90	120	180	240	B	5	15	30	60	90	120	180	240
Mean	3.7	3.7	3.7	6.5*	6.3*	5.8*	3.7	4.0	3.7	1.0	1.0	1.5	1.3	2.2*	1.8	1.8	1.2	1.0	0.5	0.7	0.8	1.3	1.8*	1.0	0.8	0.7	0.8
sd	0.8	0.8	0.5	1.0	0.8	1.2	0.8	0.9	0.5	0.9	0.6	0.8	0.5	0.4	0.4	0.8	0.4	0.6	0.8	0.8	0.4	1.0	0.5	0.6	0.8	0.8	0.8

**Table 2:** Heart and respiratory rate after single-dose administration of prucalopride; SD = standard deviation.

Time (mins)	HR (mean ± SD)	RR (mean ± SD)
B	34.3 ± 5.2	12.7 ± 1.2
5	35.0 ± 5.0	12.7 ± 1.8
15	34.5 ± 4.2	13.3 ± 1.2
30	34.2 ± 4.4	13.7 ± 1.9
60	34.3 ± 3.9	12.8 ± 1.5
90	34.3 ± 3.9	12.8 ± 1.2
120	34.8 ± 4.3	13.2 ± 1.2
180	34.5 ± 3.7	13.7 ± 0.8
240	33.7 ± 2.9	12.8 ± 1.7

HR: heart rate; RR: respiratory rate (p = n.s. for all time intervals for both variables).
